# Agriculture systems dataset in rural communities of Hidalgo state, Mexico

**DOI:** 10.1016/j.dib.2023.108918

**Published:** 2023-01-18

**Authors:** Yaxk'in Coronado

**Affiliations:** Centro de Investigación en Alimentacion y Desarollo Unidad Hidalgo, CIAD-URH CONACYT, Mexico

**Keywords:** Agricultural systems, Sustainability, Living spaces, Rural socioeconomic

## Abstract

The agricultural systems are commonly underrepresented by the decision making, due to difficulties obtaining data in the rural work, so then we collect data corresponding to a socio-economic and environmental survey applied to understand the relations between agricultural production, household economy, small stakeholders family structure, corn production and associated crops, and producers siblings acquiring sites of foods, edible plants and animals. The information was obtained by a survey applied to a rural population with high and very high poverty conditions into municipalities of Ixmiquilpan and El Cardonal in Hidalgo state at the center of Mexico. We performed a statistical analysis to identify the main socio-economic, agricultural and household characteristics in order to show an scope of the agricultural situation and household of rural agricultural communities in Hidalgo. This information allows us to design a recommendation for access to financial support or technical assistance in order to improve their production according to their sociocultural conditions. The data include socioeconomic values that are statistically representative of the official governmental values, proving the sufficiency of data and reproducible form in order to compare this data to other agricultural systems reported in Mexico. In the future a temporal comparison with federal data is desirable.

**Specifications Table**

**Table:** 

Subject	Agronomy and crop seed / Applied Machine Learning
Specific subject area	Models for sustainability.
Type of data	-Table -Image -Chart -Graph -Figure
How data were acquired	Survey with Kobotoolbox, and plotly library in Python.
Data format	-Raw data from the surveys, -Analyzed with Pandas phyton. -Filtered by municipality -Plotted plotly.
Parameters for data collection	The data consider a population with a low income with a system of corn crops and associated crops, known as milpa. Additionally the families was interviewed for a global context of the
Description of data collection	The data collected involve four main categories, socio economic data related to the producer, family structure, Agricultural production and sites of nourishment. The data are linked by the ID producer to the different siblings, main and associated crops and production associated to nourishment sites in the municipalities studied.[Provide a brief description of how these data were collected. Max 600 characters]
Data source location	Institution: CIAD City/Town/Region: Hidalgo/Ixmiquilpan and Cardonal/ Alto Mezquital. Country: Mexico Latitude and longitude: Primary data sources: Survey developed by CIAD
Data accessibility	Repository name: Github - Agriculture_dataset Direct URL to data: https://github.com/yaxastro3000/Agriculture_dataset/tree/master Instructions for accessing these data: The access to the data is in the folder Data.

## Value of the Data

• The data set provides primary agricultural, socioeconomic and cultural data from smallholder farmers in conditions of extreme poverty and highland arid regions, in two water systems: irrigation and rainfed for polyculture and monoculture of corn; useful to identify the relationships between the household socio-economics and management of their livestock resources fundamental to design strategies for rural regions for their territorial development.
• The dataset can be used by the scientific communities and smallholder farmers to understand and identify the critical points in the management of the livestock resources as well can be used by educators, agricultural and social science researchers with the farmers to model agricultural scenarios to improve the decision support of the smallholder farmers.
• The data are allowed to be used to search relationships between agricultural rainfed and irrigated systems in rural conditions in dryland conditions, socio economic conditions of the households of farmers and their relationship with the strategies used to manage the natural resources and model the effects of the agricultural practices in dryland conditions to the farmers household, economy and community.

## 1 Data Description

The agricultural lands in drought conditions take an important relevance now with the recent global climate change affecting the crops, increasing the desertification of agricultural lands and reducing the yield. The common solutions in developing countries are on one hand, the use of wastewater for agricultural irrigation systems, making them an increase over agricultural productivity but an ecological disaster in a long term [1], but on the other hand, the crop adaptation to the rainfed agricultural systems to the climate change in these regions [2], has a low productivity with cultural practices of maize conservation, leading to a environmental sustainability compared to the irrigation system. These contrasting conditions are privileged in Mexico at the central region, in particular the Hidalgo state has a region called El alto Mezquital where two co-spatial municipalities Ixmiquilpan and El Cardonal share the same culture, known as the Hñähñu region. the first one with mainly irrigation systems for their crops and the second one with predominant rainfed agriculture in semi desert regions, combination of their crops with a diversity of cactus, maguey and arid plants.

The data collected from both municipalities allow us to compare irrigation and rainfed systems in the same geographical region for semi-desert lands, understanding the main variables that affect the agriculture in conditions of desertification expected for the global climate change in rural areas.

The agricultural data from the farmers is one of the most difficult to collect due to the distance from the urban regions and the rural infrastructure, most of the studies involve a personal interview without electronic devices that support the recollection of data [3]. The value of agricultural data helps us to understand the social, economic and environmental situation of the smallholder farmers, and identify the main clues about the effect of climate change in the production of nourishment. The arid lands in special has an impact of the climate change.

These data are collected as a primary source from 115 farmers in the region of El Alto Mezquital Hidalgo with corn crops in monoculture and polyculture, mainly maize crop with other crops, known as “Milpa”, in two municipalities, Ixmiquilpan and Cardonal,the first one mainly in irrigation systems and the second one with rainfed. The data collected includes socioeconomic data from the farmer and their household, their food supplies and areas or regions where the household obtains food for the household, these regions are known as the living space, as well a quantification of the annual recollection or production for food self-sufficiency.

The data is divided in six sections: farmers general data, farmers family data, land and harvest or recollected products, living space, activities in the living space and products from the living space. In Table 1 describes each one of the variables, some of them have units.

**Table 1:** Variables included in the data file, including description and units.

Variable	Description	Unit
Section 1: farmers general data.		

Farmer_ID	Unique identifier	
Municipality	Name of the municipality	
Age	Age of the farmer	years
Gender	Farmer Gender	
Activities	Main and secondary activities of farmers	
Mem_fam	Number of family members in the household	
Land	Number of land used by the family	
Num_Liv	Number of living spaces farmer visit	

Sections 2: Family		

Family_ID	Unique identifier for family member.	
Relationship	Relationship with the farmer	
Age_fam	Age of the sibling farmer	years
Scholarship	Education level of the farmer sibling	
Act_fam	Main and secondary activities of sibling farmer	

Section 3: Land		

Land_ID	Unique identifier for land used by the farmer	
Extension	Area of agricultural plot	Hectare
Prop_status	Property status of the agricultural plot	
Use_plot	Type of use of land (agricultural or livestock)	
Type_plot	Type of land (irrigation or rainfed)	
Prod_plot	Type of production plot (Monoculture, polyculture, etc)	
Crop	The crop is indicated in the column as (corn, alfalfa, squash, etc), cultivation use (Self-consumption, sale or both of them).	

Section 4: Living space		

Living_space_ID	Unique identifier for the living space used by the farmer.	
Liv_space	Name of the living space (Backyard, Tianguis, Milpa, Mountain)	
Act_liv	Number of activities developed in the living space	

Section 5: Activities		

Activity_ID	Unique identifier for activity developed in the living space	
Act_name	Name of the activities developed in the living space.	
Prod_num	Number of products obtained in the living space	

Section 6: Products		

Product_ID	Unique identifier for the product obtained in the living space	
Prod_name	Name of the product obtained in the living space.	
Vol_prod	Amount or volume of the product obtained in the living space.	
Unit_prod	Unit of measure of product obtained in the living space (kilos, bunch, bundle, etc.)	
Liv_per	Percentage of time spend by the family in the living space per product.	

The data for this study can be found in the link: [4], in order to structure the data between the different sections, we use the value of Table 1, Section 1: ID_farmer, in tables called family, land and living space. From living space table we use the value: Living_space_ID in a table called activities. Finally in the activity table we use activity_ID value in table products. These IDs allow us to structure the data in a tree form, identifying each smallholder farmer with their family, land products and living space products.

The living space table is classified in five areas, Milpa or vegetable garden (orchid), backyard system, market and hills or mountain, each living space area has associated activities, like hunting, recollection, buy and sell, etc. Finally each activity has associated the products obtained in the different living space and the time spent in these activities.

The data was collected directly from the farmers via a survey implemented via the tool Kobotoolbox [5], using the web platform and the app Kobocollect for the field work in the communities with the farmers. The survey contains around 300 questions with conditional conditions, according to the different products or crops harvested by the farmer . The data was cleaned and pre-process in order to anonymously access the data, in order to obtain 25 attributes from the 115 records.

The farmers data collected in the survey were selected with restrictions to the socio-economic status of farmers that are in extreme and high levels of poverty according to the National Council for the Evaluation of Social Development Policy (CONEVAL by their acronomys in Spanish) Survey 2015 [6]. The farmers need to belong a indigenous community, and have at least one parcel with maize or with other crops.

The next section we describe the most general data of the survey in order to see the relevance to the socioeconomic situation of indigenous communities at family level of farmers in the Alto Mezquital, provide us valuable information on the rural units, in order to design better strategies and government programs that drive regional development.

**Fig. 1:**
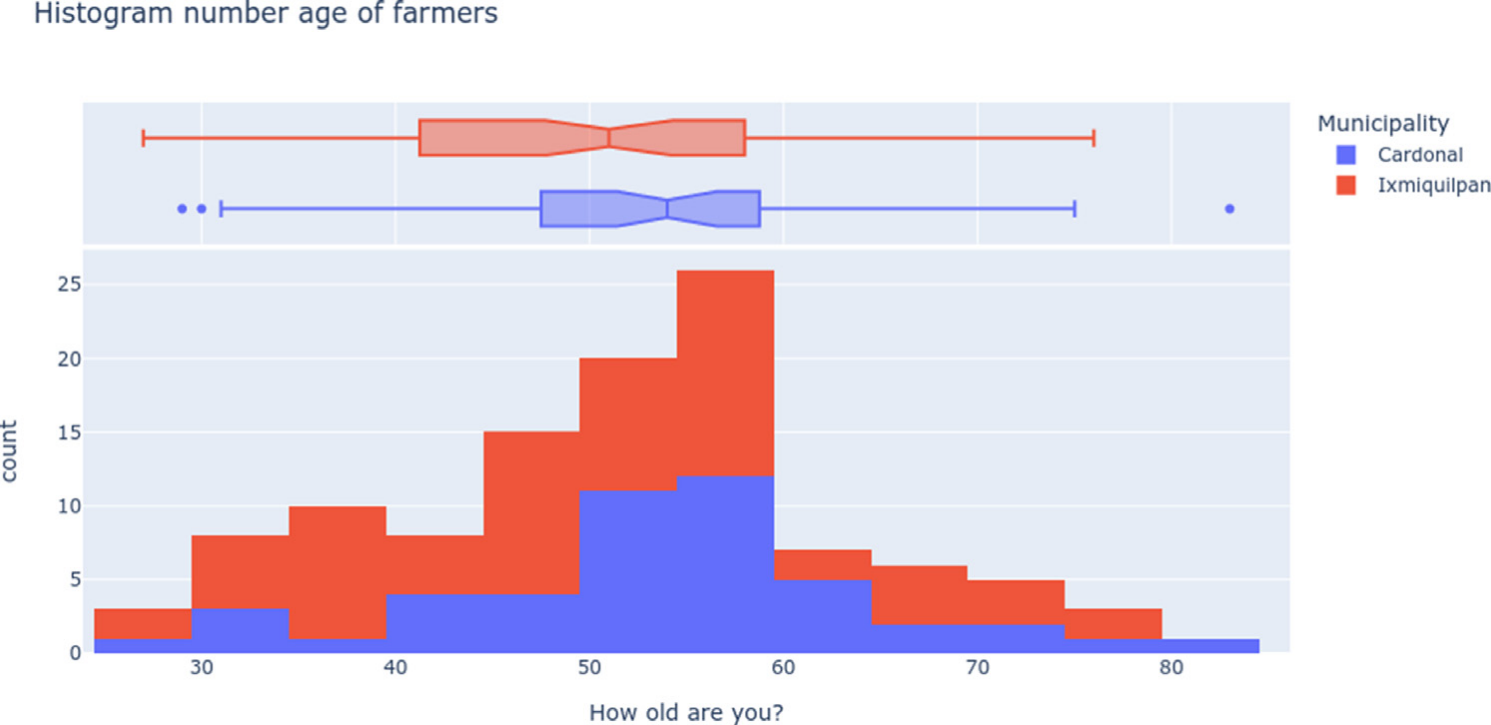
Histogram and box plot distribution of the farmers’ ages from the municipalities of Ixmiquilpan and Cardonal. The age of the farmers tends to a middle age between 50 and 60 years, with an asymmetric distribution tending to older farmers’ age. Therefore, the age representation shows a gap between 30 and 40 years for Cardonal indicating an unlinked between generations of farmers by the age.

**Fig. 2:**
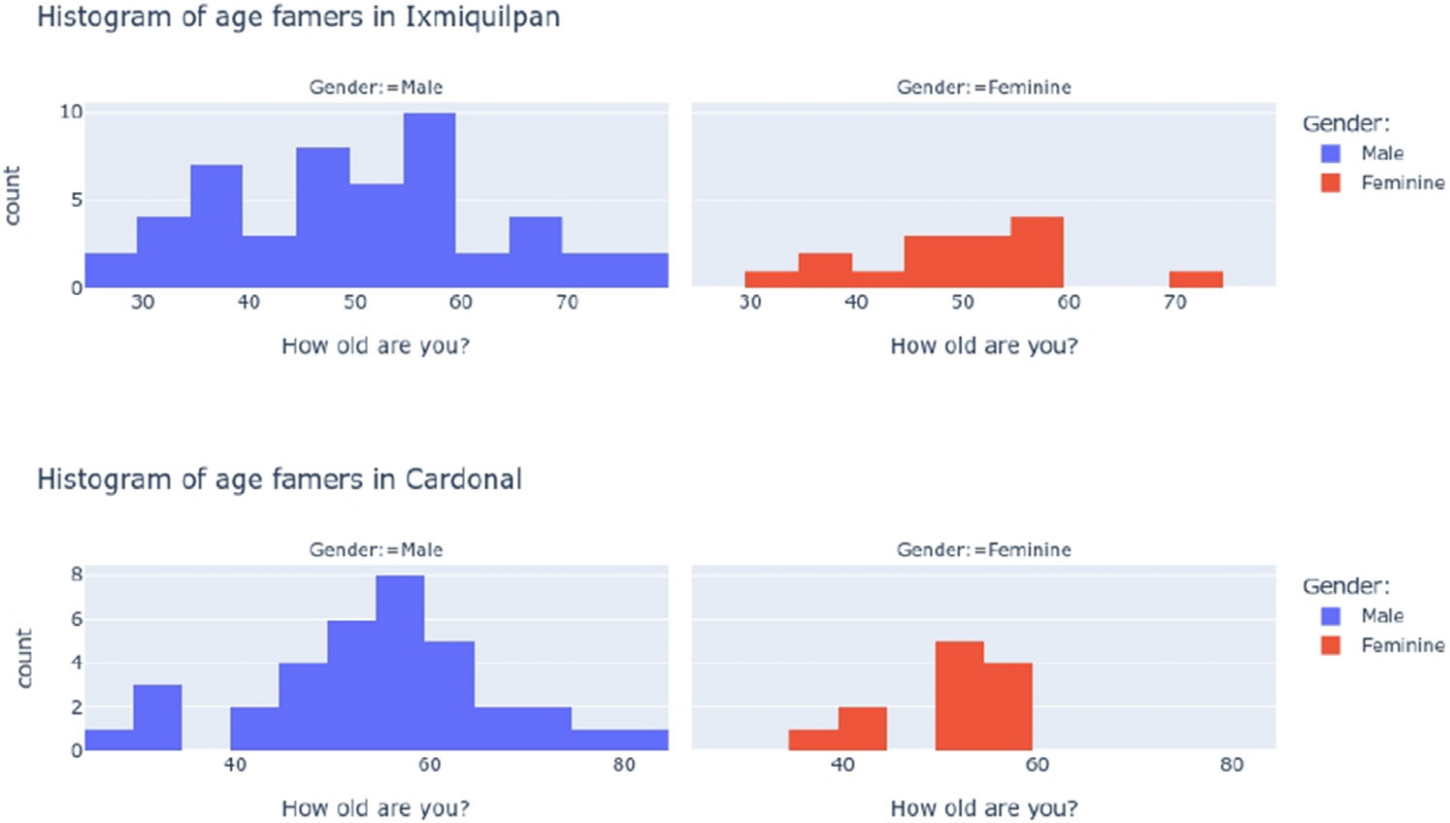
Histogram of age farmers classified by gender in each municipality.Therefore the female farmers tend to be a normal distribution for Ixmiquilpan and a concentrated distribution for Cardonal. Both distributions for male farmers dominate as a nearly normal distribution.

The general characterization of the farmer includes the age distribution shown in the Fig. 1, indicating a tendency of producers over 50 years, and few out layers for the municipality of El Cardonal to younger ages. Therefore gender is an important factor to explore, so we present the histogram of age classified for gender and municipality in the Fig. 2, with an important presence of female farmers in agricultural activities.

**Fig. 3:**
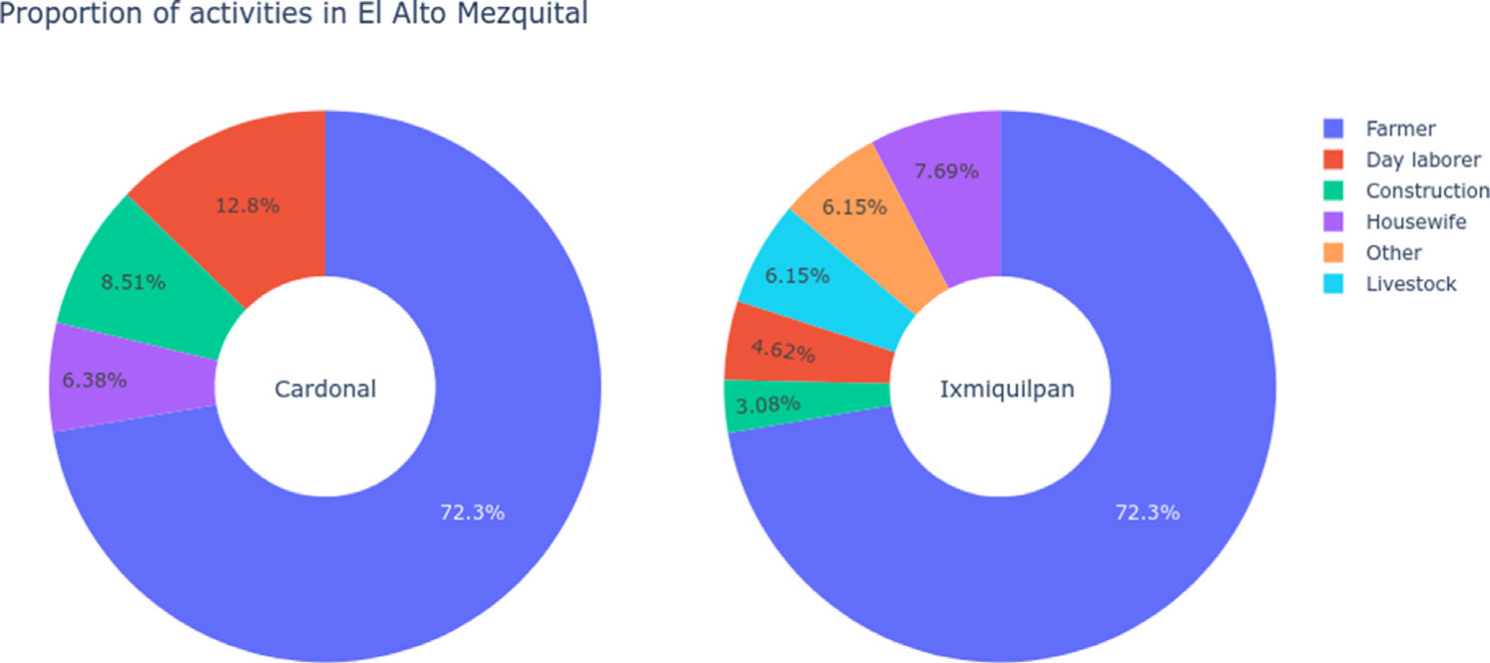
Activities developed by the farmers in El Alto Mezquital, the main activity is a farmer, but a 25 percent represent activities like day laborer, construction, housewife and other.

In order to quantify the predominance of agricultural activities the distribution of these developed by the farmer per municipality are shown in the Fig. 3, with a quarter of their activities corresponding to construction, household activities and commercial activities.

**Fig. 4:**
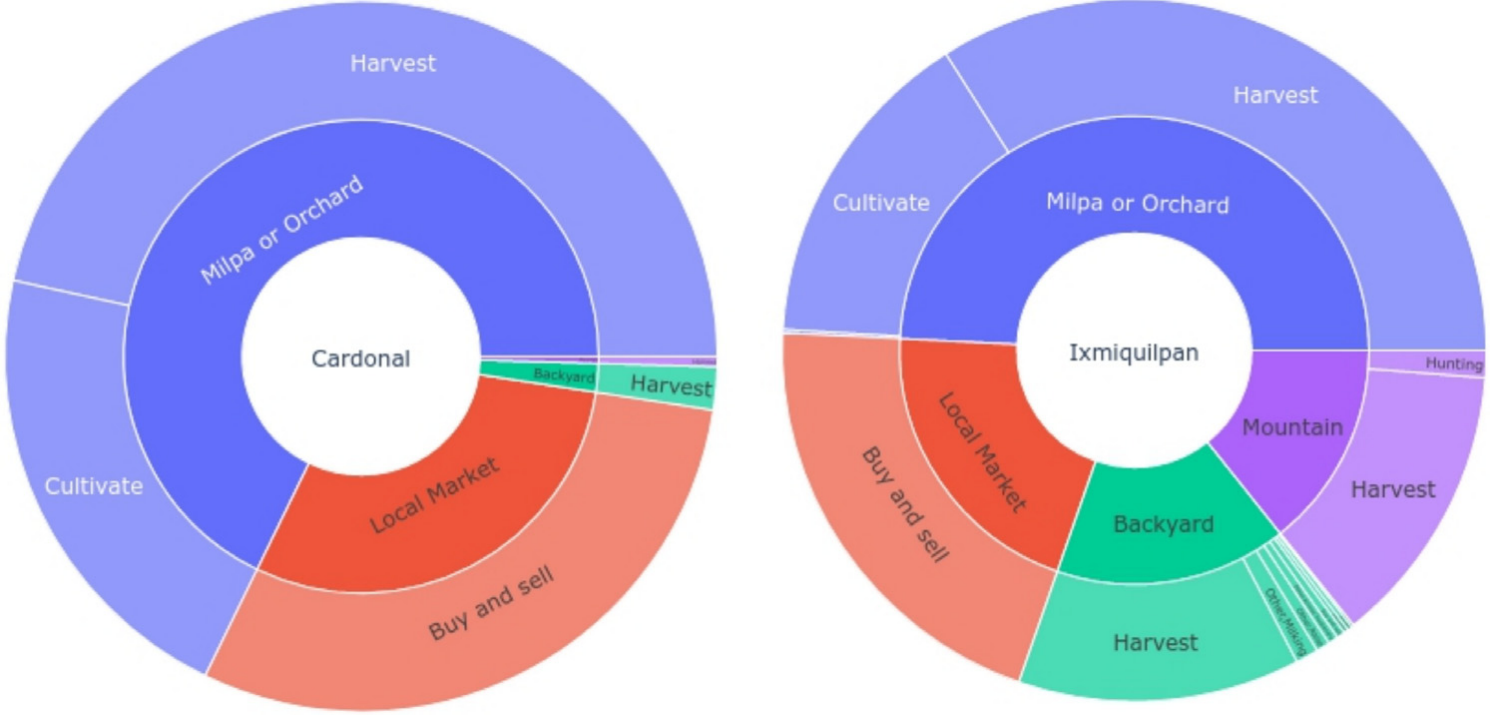
Distribution of living space and activity developed by the farmers in each municipality. Therefore the Cardonal municipality has almost three quarters of their activities in the milpa or orchard while in Ixmiquilpan this living space covers a half of the activities. On one hand in Ixmiquilpan note a regular distribution for the other living spaces, on other hand in Cardonal only the local market has an important relevance.

The nourishment of the farmers are connected with the living space, these sites are classified in five areas, in particular the Mezquital valley only present four of these sites and the principal is Milpa or orchid with main activity harvest followed by cultivate, in the Fig. 4. Additionally we present the figures separated by municipality, it is clear that Ixmiquilpan has a diversity of sites for the living spaces, having an extensive use of the territory, and the main concentration of sites in Cardonal are the milpa and local market. Therefore it is important to note that the hunting activity in Cardonal is banned due to conservation and ecological regulations.

**Fig. 5:**
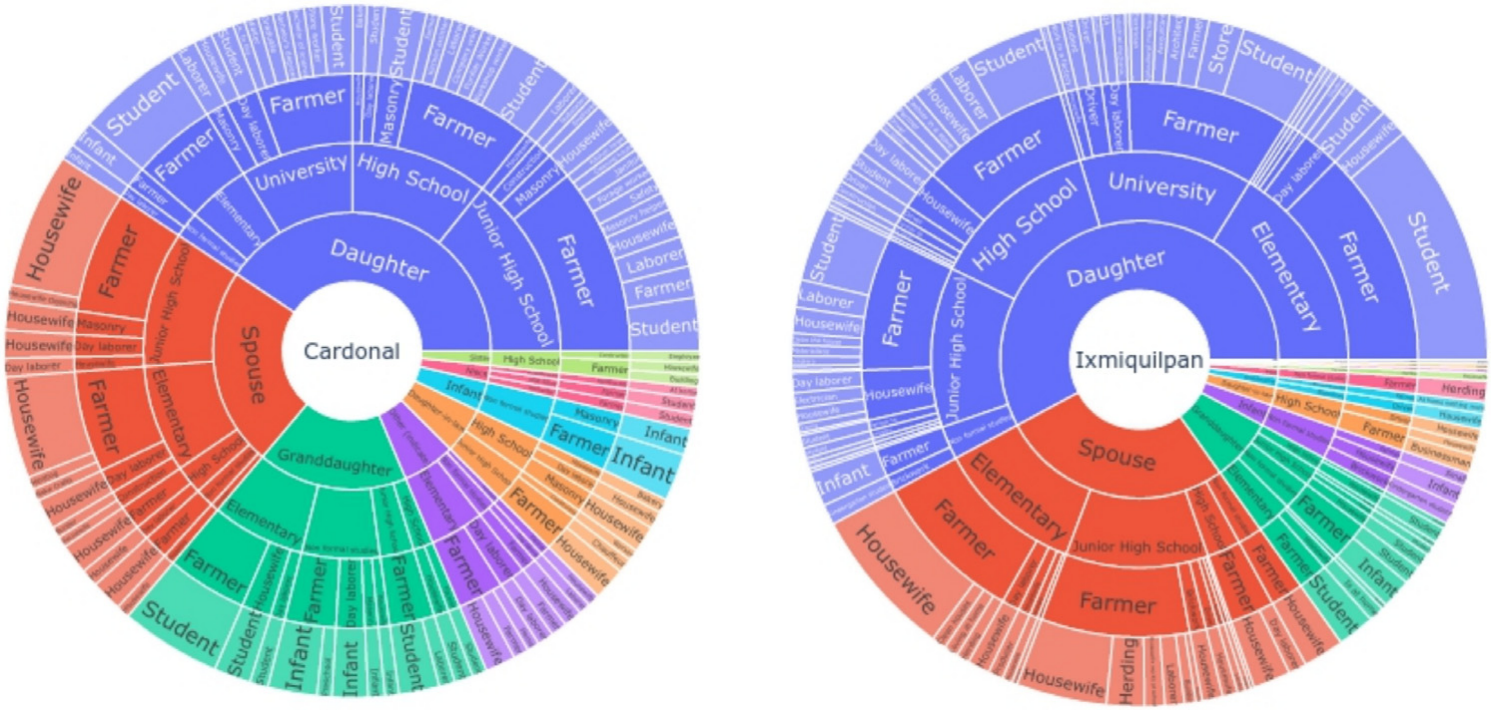
Distribution of relationship of the family with the farmer, educational level of siblings, main activity of the farmer, and main activity of the sibling, for the two municipalities. The main composition of the families are the spouse and daughter for both municipalities followed by a granddaughter in Cardonal with almost 75 percent of farmers with this structure, and extentded diversity of relationship in the same household in Ixmiquilpan.

The family conditions are summarized in the Fig. 5 with the distribution of relationship with the farmer, scholarship of the siblings, main activity of the farmer and main activity developed by the siblings showing a predominance of household composed by the farmer and spouse and children in scholar age.

**Fig. 6:**
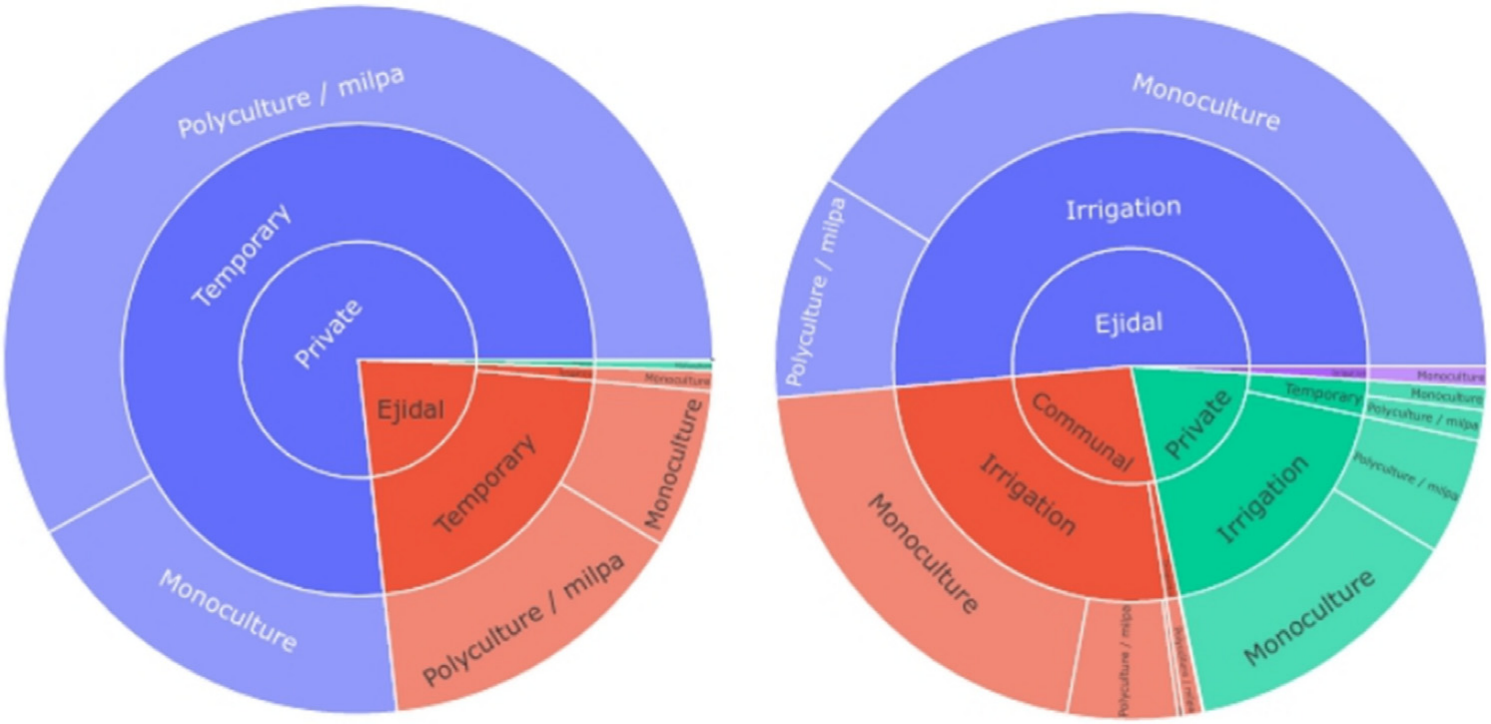
Distribution of regime of property of agricultural plots, production system, and type of crops, at the left hand side the Cardonal Municipality and the right hand side correspond to Ixmiquilpan municipality. Therefore Cardonal municipality has mainly private properties with rainfed or temporally agriculture in polyculture or milpa while in Ixmiquilpan the regime of property is distributed between Ejidal and communal, with irrigated systems and mainly monoculture crops.

The distribution of land in property regime and extension are presented in the Fig. 6, with a predominance of private properties for El Cardonal, in temporary regime, and a balance of properties in Ixmiquilpan with mainly irrigation systems.

**Fig. 7:**
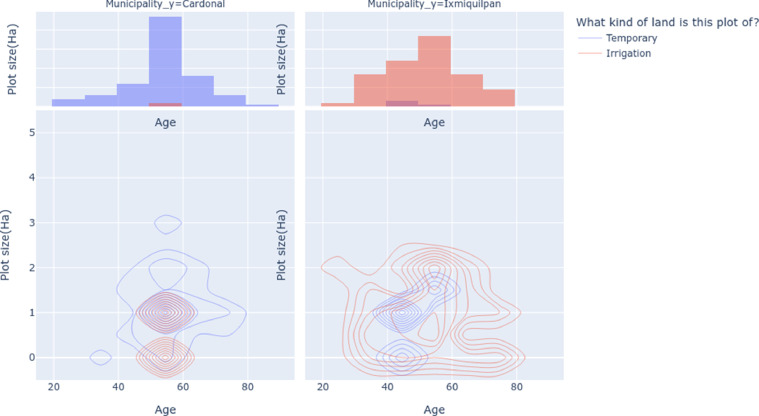
Distribution diagram of the age farmer versus plot size in hectares for temporary and irrigation agricultural systems. The dispersion of irrigation systems in Ixmiquilpan are between 0 and 2 hectare mainly and the age of farmers from 20 to 80 years old. Cardonal distribution is concentrated between farmers of 40 to 60 years old and plot size of one hectare and nearly zero for irrigation systems.

In reference to the agricultural system managed by the farmers and the extension of the land per municipality we see in the Fig. 7, that the main system of agricultural production for Ixmiquilpan is irrigation and rainfed for Cardonal. Respect to the extension of the land the rainfed systems has an extension from 1 to 5 hectares in Cardonal and for the irrigation systems a concentration in 1 to 2 hectare for ixmiquilpan.

**Fig. 8:**
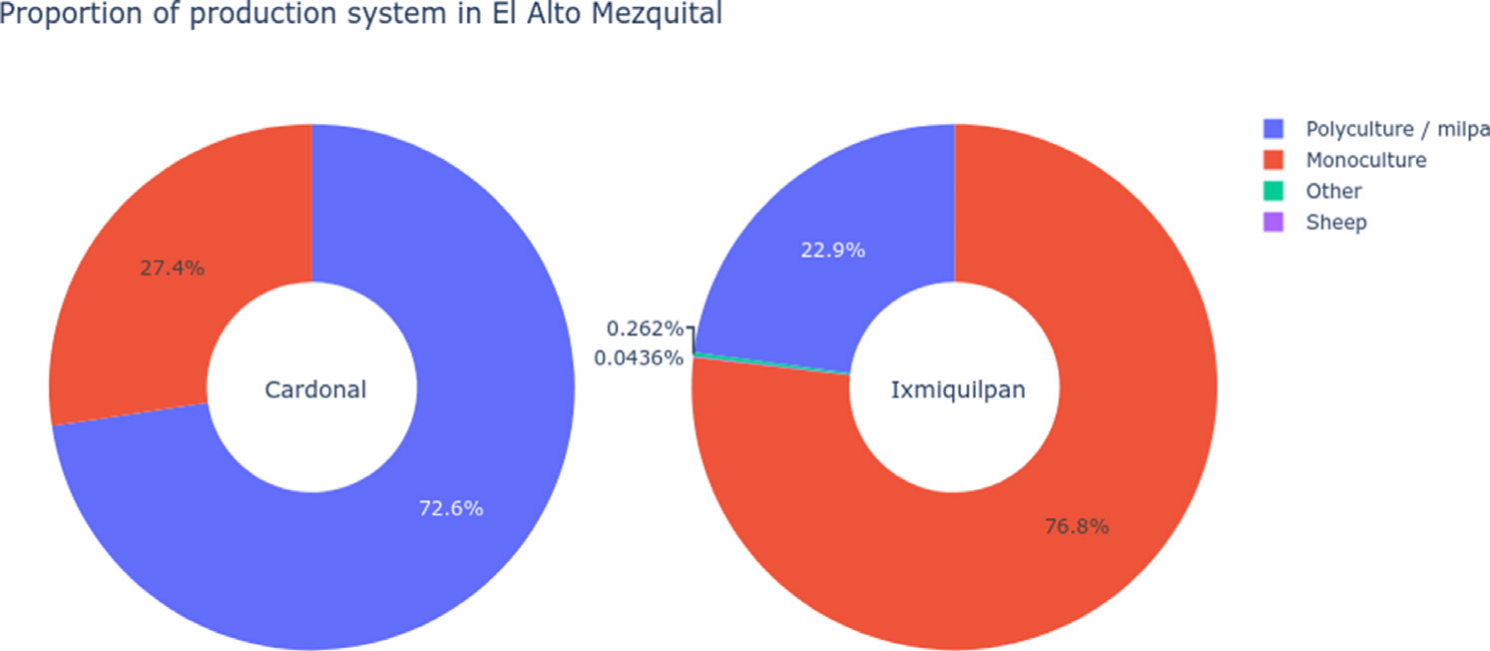
Distribution of the agricultural systems in El Alto Mezquital, between the two municipalities the main differences are in the system of monoculture dominated in Ixmiquilpan versus the polyculture system of milpa dominated in Cardonal.

Follow the agricultural system of both municipalities, the production have important differences, with almost 75% of monoculture for Ixmiquilpan and 25% for Cardonal, and 25% of polyculture in Ixmiquilpan and 75% in Cardonal, for more details see Fig. 8.

**Fig. 9:**
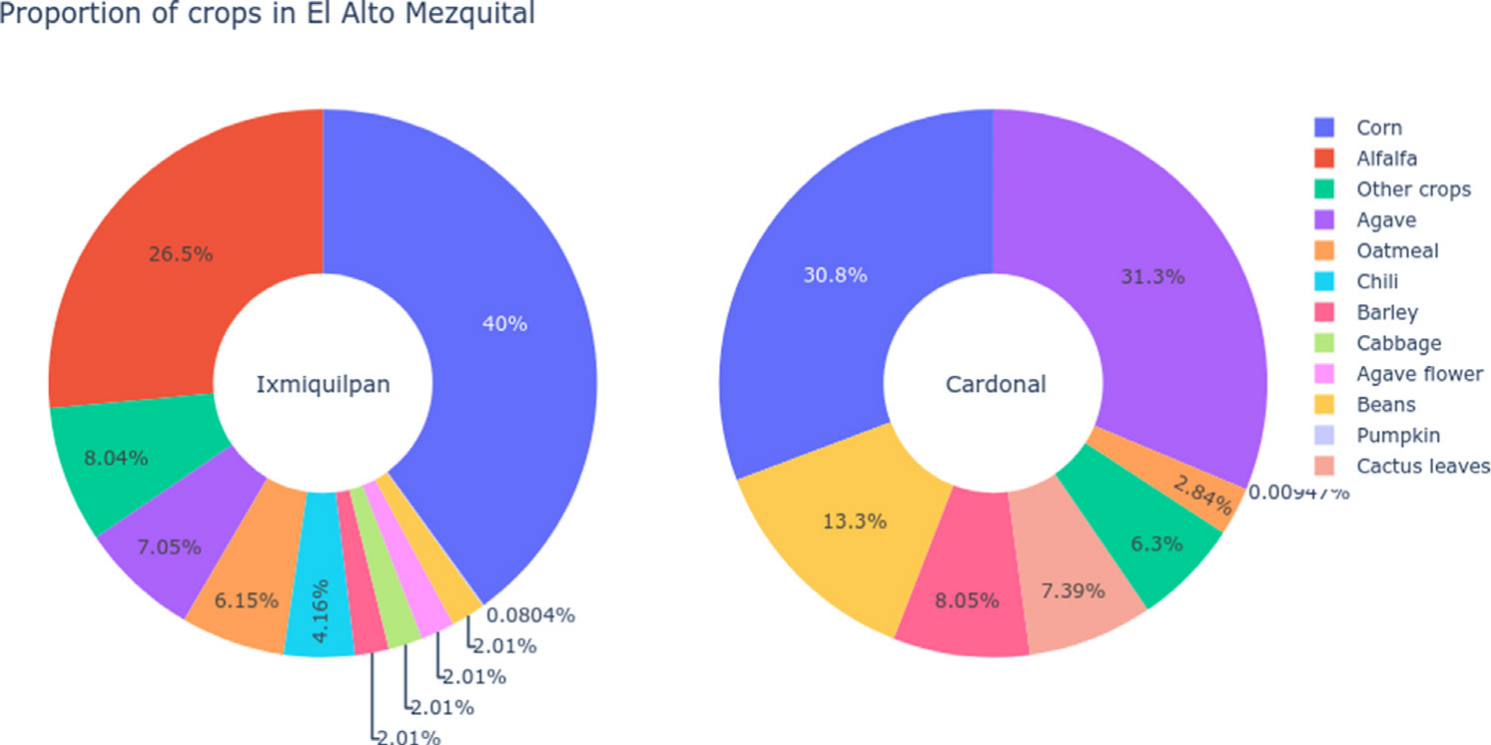
Distribution of crops cultivated or used in El Alto Mezquital. The main crops in Ixmiquilpan correspond to corn and alfalfa in irrigated systems versus El Cardonal; the polyculture system is composed mainly of corn, maguey, beans. The diversity of crops in Cardonal responds to different uses like the maguey flower in their gastronomy.

Therefore, the cultivation and harvest of crops are concentrated to two main crops per municipality, in Ixmiquilpan the corn and alfalfa are the predominant with monoculture, for Cardonal the main crops are corn and maguey, follow by beans, see Fig. 9 for more details.

The main constriction to the data are the socio-economic situation in extreme and high level 2015 [5] of poverty in indigenous communities according, selecting only communities in these range status. Additionally we reproduce the socio-economic distributions for the age, scholarship and family structure from Mexico's National Institute of Statistics and Geography (INEGI by their acronym in spanish), using the data of the intercensal survey 2015 [7] and Census of population and housing in 2020 [8] (Table 2).

**Table 2:** Comparison between the socio-economic parameters in percentage per municipality from government and our survey.

Parameter	INEGI^1^	Cardonal	INEGI^1^	Ixmiquilpan
**Education level**				
Elementary and Junior High School	58.0	57.5	52.6	61.6
High School	19.1	17.8	22.7	17.83
University	13.9	10.27	19	12.97
Non formal studies.	9	15	5.6	7.5
**Non economic active population**				
Students	33.7	26.5	38.4	39.4
Houseworking	43.7	36.05	40.3	33.5
**Family structure**	INEGI^2^	Cardonal families	INEGI^2^	Ixmiquilpan families.
Nuclear	69.7	45	69.7	60
Extensive	30.3	55	30.3	40

1 Data from 2020 Census of population and housing 2. Inter censal survey 2015. The terms of use of INEGI data, are in free use of information [9], and data avaiable in english version [4].

The supplementary data contain the information described in Table 1, divided in the six sections shown in excel format, each table has their corresponding identification. Allow us to study the data as a tree structure for the tables depending on the general farmer data. Additionally to the database we include the survey applied to the farmers.

## 2 Experimental Design, Materials and Methods

The data were collected as part of a investigation program for the characterization of indigenous communities in the Alto Mezquital, Hidalgo, Mexico, in extreme and high level of poverty, with a main crop maize, the data is a mix between the small-scale production, low input and self-consumption for rainfed and irrigation systems with a median scale production, for mainly commercial purpose. The survey includes the analysis of the household economy, activities and sites where surrounding, the farmers for recollection and harvest food for their families, extension of the plots and diversity of crops planted or maintained in the agricultural fields as an unit, denominated family production unit.

Previously to the survey implementation, we estimate the population expected, according to restrictions of communities in high and extremely high poverty levels in agreement with the National Council for the Evaluation of Social Development Policy (CONEVAL), since the population of the two municipalities are indigenous farmers, that belong to the Otomi region, represents the 5.6% of the total population of Hidalgo state [10], our study used the equation following [11]:

(1) $$n=\frac{{\left[Z_{\alpha /2}\right]}^{2}\hat{p}\hat{q}}{E^{2}}$$

Where n = sample size, $Z_{\alpha /2}$ is the value that split the area α/2 for the right tail of the normal distribution (for a confidence level of 95%, α=0.05 $Z_{\alpha /2}=1.96$), $\hat{p}$ is the ratio of the population studied respect to the total population (for population of 5.6%, so $\hat{p}\;=\;0.056$), $\hat{q}\;=1-\hat{p}$ ($\hat{q}=0.949$) and E^2^ is the margin of error (0.05). A sample size of 81 was calculated. Following the above, the target sample size for this study was determinated to be around 100. Data collection was separated to be at least 50 interviews for each municipality and communities identified by CONEVAL in high and extremely high poverty.

The survey was constructed using the software Kobotoolbox for the implementation and data recollection, including the six sections with conditionals and loops in the survey to optimize the data recollection and minimize the data correction with a verification in the field and a verification of the information with the interviewer.

The implementation of the survey in field work, include the use Kobocollect app for android cellphone that allow us to collect data via offline and recorder the geo-localization including media data as photos from the agricultural plot, saved in the point of collection and after in an stable connection to wireless internet send the information to the servers.

Initiality the data was reviewed to fill the criteria of localization, polyculture land and randomness of the interviewers selecting farmers that are not familiar with others in the community. Additionally we review the area as a representative of the drylands area with similar climate conditions with mediterranean zones in Spain and African continent. The indigenous population of the zone

We implement a cross check verification of the data with the interviewer normalizing units and size of the plots with an inspection of the local units and factor of convertion. Therefore the qualitative data was classified and reorganized into categories in order to simplify the analysis. Most of these actions was do it with the software Orange Data Mining [12], identifying the main distributions of the data and statistical values of the region, the main finding in the data was the difference between the municipalities of Cardonal and Ixmiquilpan in their production systems. The analysis presented in the figures was worked in python libraries like pandas and plotly to explore the relationship between the data and describe the statistics of the dataset.

The survey was implemented in 2019 with a series of socioeconomic data like age, municipality and communities interview, we show the reproducibility of the data at least in the distributions of socioeconomic data available from governmental sources, with a comparative between the INEGI data 2015 and 2020 reproducing the expected percentages numbers of education level of families, The main two non active working population and family structure in the range of error according to the 95% of confidence level.

The initial recollection of information was made through the app Kobocollect, applied to the farmers to provide information about the production of polyculture system Milpa, the family structure, farmer's socioeconomic data and living spaces, sites where around the farmer's recollect and harvest food for the family.

This dataset is the first effort to bring a detailed relationship between agricultural communities from indigenous populations with their socioeconomic, environmental, household economy and cultural practices to arid land regions in order to identify the best practices to face the challenges of the global climate change in agriculture.

## Ethics Statement

This work involved human subjects, to the extent that the data collected included spatial location of farmer's fields, information related to agronomic activities, familiar activities, and income information. Therefore, a relevant informed consent was obtained by informants through the data collection system, describing the use of the data, as well as the sharing code for third parties.

Farmers, family farmers, and legal guardians or parents of under age participants agreed to provide information with which CIAD could (a) generate and disseminate statistical information related to social, agronomic and environmental scientific research, after anonymizing the data obtained; (b) generate information and/or studies regarding agriculture or related disciplines, including uses and practices in agriculture, socioeconomic conditions and nourishment security level in certain population, zone or region.

## CRediT authorship contribution statement

**Yaxk'in Coronado:** Conceptualization, Methodology, Software, Data curation, Writing – original draft, Visualization, Investigation, Supervision, Validation, Writing – review & editing.

## Declaration of Competing Interest

The authors declare that they have no known competing financial interests or personal relationships which have or could be perceived to have influenced the work reported in this article.

## Data Availability

Agriultural data from Alto Mezquital (Original data) (Github).
